# Effects of cancer-associated fibroblasts on the migration and invasion abilities of SGC-7901 gastric cancer cells

**DOI:** 10.3892/ol.2012.1023

**Published:** 2012-11-09

**Authors:** CHENGYI HU, ZHENGCAI WANG, LILI ZHAI, MILING YANG, LIHUI SHAN, CUICUI CHAI, MING LIU, LIFENG WANG

**Affiliations:** 1 Department of Pathology, The Fourth Hospital of Harbin Medical University, Harbin 150001;; 2 Department of Pathology, Xinhua Hospital Affiliated to Shanghai Jiaotong University School of Medicine, Shanghai 200092, P.R. China

**Keywords:** gastric cancer, cancer-associated fibroblasts, SGC-7901, gastric cancer cells

## Abstract

The aim of the current study was to investigate the correlation of cancer-associated fibroblasts (CAFs) with the migration and invasion abilities of gastric cancer cells. Gastric CAFs were grown in primary cultures. The *in vitro* model of interaction of SGC7901 gastric cancer cells with gastric CAFs was established by the use of Transwell co-culture cells to analyze the influence of CAFs on the migration and invasion abilities of SGC7901 cells. The results revealed that i) human gastric CAFs highly expressed vimentin, fibroblast-activated protein and smooth muscle actin in the *in vitro* passage culture process; ii) the migration and invasion ability of SGC-7901 cells in the CAF-conditioned medium group (98.67±13.49, 34.40±4.63) were significantly higher compared to those of the DMEM group without serum (78.47±10.59, 26.93±3.99; P<0.01). The interactions of CAFs and the extracellular matrix with SGC-7901 cells may significantly increase the migration and invasion abilities of SGC-7901 cells.

## Introduction

Gastric cancer involves tumors of the digestive system and has the highest incidence and mortality rate in China (1). The overall 5-year mortality rate of patients is more than 80% worldwide. Gastric cancer has the second highest mortality rate of all types of cancer in the world (2) and therefore seriously threatens human health. Currently, it is thought that tumor stromas play a significant regulatory role in the balance of the tumor-host interface microenvironment (3,4). As stroma cells are non-variant cells and their genetic compositions are relatively stable, they are easily studied and have gradually become a significant focus of research (5). Cancer-associated fibroblasts (CAFs) are the main cellular component of tumor stromas. Previous research has suggested that CAFs have different phenotypes and biological characteristics from normal fibroblasts (NFs) and alterations to these phenotypes are mostly associated with the infiltration and metastasis of tumors (6,7). However, at present little is known about the role of CAFs in gastric cancer invasion and metastasis. Therefore, human gastric CAFs were isolated and cultured *in vitro* to establish a model of interaction of CAFs with SGC-7901. Their influences on the migration and invasion abilities of SGC-7901 gastric cancer cells was further analyzed. This study is likely to aid in understanding the infiltration and metastasis mechanisms of gastric cancer cells and may provide a reference for clinical treatment.

## Materials and methods

### Cells

The gastric cancer cell line SGC-7901 was provided by Dr Chen Xi, Department of General Surgery, First College of Clinical Medicine, Harbin Medical University and cultured in high-sugar DMEM containing 10% fetal bovine serum in an incubator containing 5% CO_2_ at 37°C. All experimental cells were in the logarithmic growth phase.

### Primary culture of CAFs

The fresh specimen from radical correction of gastric cancer was taken from the operating room and sheared into small blocks 1 mm in diameter on an aseptic bench and then placed uniformly onto the culture face of a disposable culture flask with an elbow straw (the interval between the blocks was 5 mm). High-sugar DMEM (2 ml) containing 20% fetal bovine serum was added, the culture flask was then inverted and cultured for 1 h in the incubator containing 5% CO_2_ at 37°C. Subsequently, the culture flask was overturned. The culture liquid was replaced on the third day and then every subsequent three days. The soonest cells were seen to appear was following 5 to 7 days. After ∼2 to 3 weeks, cells covered the whole base of the flask. The enzyme digestion method was used to purify the cells. Following the first passage, the medium was replaced with high-sugar DMEM containing 10% fetal bovine serum to conduct a conventional culture. The third-generation purified cells were used to conduct preliminary identification. In addition, the 4th to 10th generations of cells were used for subsequent tests.

### Identification of human gastric CAFs

The cover glass with cells was fixed with 95% alcohol for 30 min. The first antibodies included cytokeratin, vimentin, smooth muscle actin (SMA) and fibroblast activation protein (FAP). The PV6000 general-type two step method was used according to the reagent instructions. In addition, the negative and positive control groups were set. If pale brown or brown granules appeared in the cytoplasm, the specimen was judged as positive.

### Preparation of CAF conditioned medium (FCM)

Human gastric CAFs were inoculated into a 50-ml culture flask and a conventional culture was carried out for 48 h. Subsequently, the medium was replaced with 5 ml DMEM containing 5 g/l BSA without serum to continuously culture for 48 h. The supernatant was collected, centrifuged at 1,000 rpm, filtered with a 0.22-*μ*m membrane for sterilization and stored at −20°C for use. At the same time, DMEM containing 5 g/l BSA without serum was set as the control.

### Cell scratch test

Matrigel and DMEM without serum were mixed at a ratio of 1:2 and added to a 96-well plate at 30 *μ*l/well (these processes were completed on ice). Artificially remodeled basement membrane was prepared and air dried on the super-clean bench. Subsequently, 50 *μ*l medium containing 10 g/l BSA without serum was added into each well and incubated at 37°C for 30 min in order to hydrolyze the basement membrane. SGC7901 cells and CAFs were added to a 96-well plate (10,000 cells/well) with 5 parallel samples in each group. Conventional culture was conducted until a monolayer of cells formed. Subsequently, the cells were scratched into a linear shape along the bottom of culture plate with a 10-*μ*l sterile tip head, the relative distance of the scratch area was recorded under the microscope. Subsequently, the cells were washed with the culture liquid without serum and the variously conditioned culture liquid was used to continuously culture for 72 h. In addition, the scratch widths were observed at 24, 48 and 72 h and images were captured. According to the original distance of the damaged cell area, the relative migration distance of cells was calculated. For each group, tests were repeated three times. Calculation formula: relative migration distance = 1 − (real-time scratch width/original scratch width).

### Transwell migration test

FCM and monolayer cultured CAFs were respectively added to a 24-well plate and DMEM containing 5 g/l BSA without serum was taken as the control. In each group, three repeated wells were set. An SGC-7901 cell suspension (100 *μ*l) with a density of 5.0×10^4^ cells/ml was added to a Transwell cell and a conventional culture was conducted for 24 h. After the cells were washed with distilled water and dried with air, crystal violet staining was conducted. Under the microscope, upper, lower, central, left and right 5 view fields were selected to count the number of transmembrane cells. The mean value represented the migratory cell number.

### Transwell invasion test

Similar to the migration test, Matrigel was used to cover the basement membrane of the Transwell cell prior to the test. The inoculation density of the SGC-7901 gastric cancer cells was 1×10^5^/ml. Following crystal violet staining, the transmembrane cell number was counted under the microscope.

### Statistical analysis

SPSS software was used for statistical analysis and measurement data were expressed as mean ± SD. A t-test was used to compare the mean values between the two groups, and analysis of variance was used for the comparison of mean values among more than two groups. P<0.05 was considered to indicate a statistically significant difference.

## Results

### Identification of CAFs

**Figure 1.**
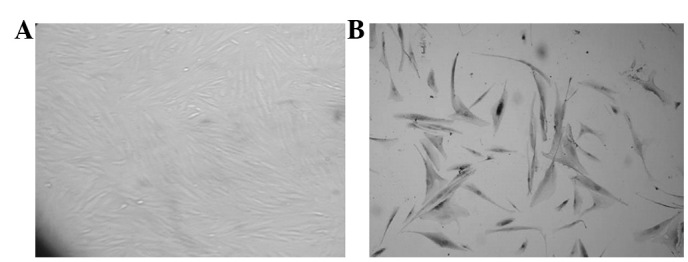
(A) Sixth-generation gastric cancer CAFs. (B) Third-generation human gastric CAFs, FAP-positive expression. CAF, cancer-associated fibroblast; FAP, fibroblast activation protein.

Inverted microscope observation revealed that purified CAFs were fibroblast-like cells, their sizes were different and their shapes presented as a fusiform or irregular triangle. Also, there were various lengths of multiple processes. The nucleus was at the center of cell body, presenting as a circular or elliptical shape. The cells were arranged in a radial or vertical shape and partial cells were disordered. Polarity, contact inhibition and density inhibition disappeared (Fig. 1A) and there were apparent overlapping phenomena. The immunocytochemical staining results revealed that the purified third-generation CAFs cells all expressed vimentin, FAP and SMA and cytokeratin was not expressed (Fig. 1B).

### CAFs promotes the transverse migration of SGC-7901 cells

**Figure 2.**
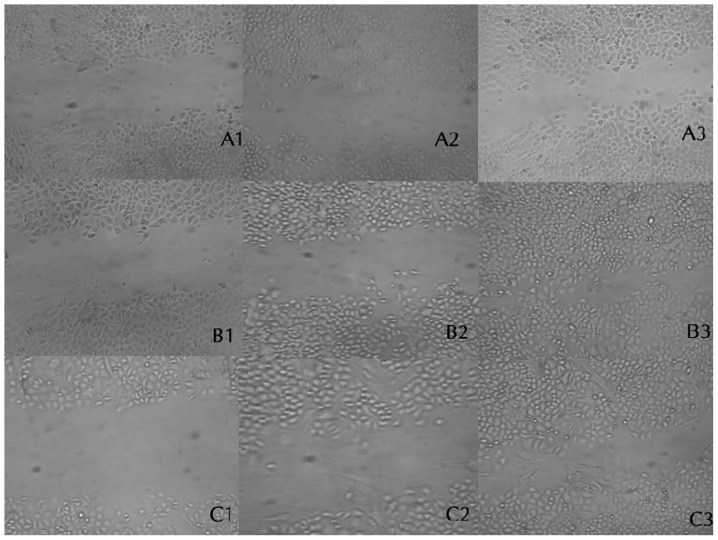
Cell scratch test results. (A1–A3) 24, 48 and 72 h of the control group, respectively; (B1–B3) 24, 48 and 72 h of the FCM group, respectively; (C1–C3) 24, 48 and 72 h of the CAFs group, respectively. CAF, cancer-associated fibroblast; FCM, cancer-associated fibroblast conditioned medium.

SGC-7901 cells and CAFs were co-cultured. SGC-7901 cells were respectively cultured in FCM or DMEM without serum for 24, 48 and 72 h. Under the inverted microscope, the relative distance of cells migrating to a damaged area was measured and calculated. CAFs had a more marked transverse migration ability than SGC-7901 cells and the migration distance of SGC-7901 cells of the CAFs and FCM group significantly increased. Therefore, CAFs promoted the transverse migration of SGC-7901 gastric cancer cells (Fig. 2, Table I).

### CAFs promotes the longitudinal migration of SGC-7901 cells

**Figure 3.**
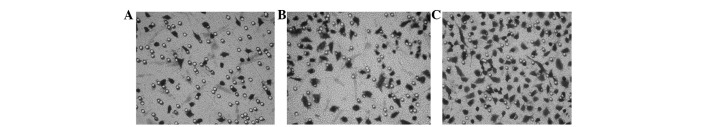
Transwell migration test. (A) Control; (B) FCM group; (C) CAFs group. FCM, cancer-associated fibroblast conditioned medium; CAF, cancer-associated fibroblast.

Transwell cells were used to co-culture CAFs and SGC-7901 cells for 24 h. The transmembrane cell number of SGC-7901 cells significantly increased, indicating that CAFs were able to promote the longitudinal migration of SGC-7901 cells and this effect was induced by FCM repeatedly (Fig. 3, Table II).

### CAFs promotes the invasion ability of SGC-7901 cells

SGC-7901 cells, CAFs and Matrigel were co-cultured and the transmembrane cell number of SGC-7901 cells significantly increased. FCM application simulated the effect of CAFs to a certain extent. Thus, CAFs promoted SGC-7901 invasion and the interaction of CAFs with SGC-7901 cells significantly promoted SGC-7901 invasion (Table II).

## Discussion

CAFs specifically refer to the myofibroblasts of the host’s malignant tumor. They were first identified in the stroma of solid tumors accompanied by connective tissue hyperplasia, including breast carcinoma and pancreatic cancer. In 1999, Olumi *et al* cultured prostate cancer stroma fibroblasts and named them CAFs (8,9). Studies have revealed that CAFs participate in the synthesis, deposition and reconstruction processes of the tumor extracellular matrix and a variety of paracrine growth factors, proteases and their inhibitors. They also induce the immunological escape of tumor cells and thus promote cytogeny and the development of tumors (10–12).

Previous research has suggested that CAFs in human gastric cancer tissues not only express fibroblast-labeled vimentin, but also the myofibroblast markers SMA and FAP. This phenotypic change of CAFs was positively correlated with a number of clinical and pathological indicators of gastric cancer (13). In the current study, an immunocytochemical technique was used to detect the expression of vimentin, FAP and SMA in human gastric CAFs cultured *in vitro*. The result revealed that human gastric CAFs cultured *in vitro* had phenotypic characteristics similar to those of CAFs *in vivo*, which was different from rat gastric CAFs isolated and cultured *in vitro*(14). This indicates that the study of human gastric CAFs may better simulate the progression of human gastric cancer.

Animal experiments have shown that in most tumor models, the interaction of tumor cells with the extracellular matrix influences the growth rate, infiltration range and distant metastasis ability of tumor cells (15). There are four steps in the invasion of cancer cells to the extracellular matrix: mutal separation of tumor cells, adhesion to matrix components, degradation of the extracellular matrix and movement of tumor cells. In this study, the cell scratch, Transwell migration and invasion tests were applied and Matrigel was used to simulate the effect of the extracellular matrix. CAFs or their conditioned medium was used to induce SGC-7901 migration or invasion and simulate the interaction of tumor cells with their surrounding CAFs and extracellular matrix. The migration and invasiveness of SGC-7901 greatly enhance under the action of CAFs. The application of FCM repeated this process, indicating that CAFs induced tumor cells to degrade the extracellular matrix by secreting soluble factors and caused infiltration and metastasis. In addition, it was revealed that under the conditions of CAFs, tumor cells and extracellular matrix co-culture, CAFs had a more marked migration ability than tumor cells. Human gastric cancer CAFs accompany invasive tumor cells *in vivo*, and they are primarily distributed at the front of neoplasm invasiveness as well as at the tumor-interstitium interface (11). Therefore, it is speculated that CAFs present invasion ability earlier than tumor cells and induce extracellular matrix reconstruction and stroma formation by secreting a variety of growth factors and proteases. The newly formed stroma damages the continuity of the normal structure and creates a pathway for the invasion of tumor cells.

CAFs promote tumor infiltration and metastasis by the following possible routes: i) sectrion of a variety of growth factors including TGF-β1 and matrix metalloproteinases and participation in tumor progression (12,16,17); ii) promotion of angiogenesis (18); iii) inhibition of the body’s antitumor immunity (19,20). In the current study, CAFs highly expressed SMA and FAP. FAP is a glycoprotein on the cell surface, with the dual activity of collagenase and dipeptidyl peptidase and it degrades multiple substrates of dipeptidase, gelatin and type - collagen in the extracellular matrix. Therefore, it is conducive to invasion of tumor cells. For tumor cells transfected with FAP, tumor formation rate and invasion ability in the nude rat body significantly increase. They also inhibit the effect of FAP in tumor tissues and significantly improve the tumor sensitivity to chemotherapy and survival rate of rats with cancer (21–23). Future research should include the effect of FAP in gastric cancer tissues, targeted inhibition of FAP and the possibility of reducing or inhibiting the influence of CAFs on the invasive ability of tumor cells. It is likely to be useful in elucidating the occurrence, development, infiltration and metastasis mechanisms of cancer cells to further clarify the cellular signal transduction mechanism associated with the interaction of CAFs with tumor cells.

This study used human gastric CAFs to establish the *in vitro* model and simulate the *in vivo* tumor microenvironment to further confirm that CAFs promote the migration and invasion of tumor cells. It also provides theoretical and practical bases for researching the mechanism of action of CAFs and their role in future tumor treatment.

## Figures and Tables

**Table I.** Scratch test results.

	Scratch width after culture (mean + SD)		
Group	24 h	48 h	72 h
CAFs	0.23±0.06^a^	0.69±0.05^a,^^b^	0.75±0.03^a^
FCM	0.24±0.05^a^	0.60±0.04^a,^^b^	0.78±0.04^a,^^b^
Control	0.11±0.03	0.18±0.05	0.27±0.04

a P<0.05 versus the control group;

b P<0.05 versus the CAFs group. CAF, cancer-associated fibroblast; FCM, cancer-associated fibroblast conditioned medium.

**Table II.** Effect of CAFs on invasiveness and migration capability of SGC7901 cells.

Group	Invasiveness experiment	Migration experiment
CAFs	98.67±13.49^a,b^	34.40±4.63^a,^^b^
FCM	78.47±10.59^a^	26.93±3.99^a^
Control	40.54±12.55	13.33±9.65

a P<0.01 versus the control group;

b P<0.01 versus the FCM group. CAF, cancer-associated fibroblast; FCM, cancer-associated fibroblast conditioned medium.
